# Transcriptomic Meta-Analysis of Multiple Sclerosis and Its Experimental Models

**DOI:** 10.1371/journal.pone.0086643

**Published:** 2014-01-27

**Authors:** Barbara B. R. Raddatz, Florian Hansmann, Ingo Spitzbarth, Arno Kalkuhl, Ulrich Deschl, Wolfgang Baumgärtner, Reiner Ulrich

**Affiliations:** 1 Department of Pathology, University of Veterinary Medicine Hannover, Hannover, Germany; 2 Center for Systems Neuroscience, University of Veterinary Medicine Hannover, Hannover, Germany; 3 Department of Non-Clinical Drug Safety, Boehringer Ingelheim Pharma GmbH&Co KG, Biberach (Riß), Germany; Hannover Medical School, Germany

## Abstract

**Background:**

Multiple microarray analyses of multiple sclerosis (MS) and its experimental models have been published in the last years.

**Objective:**

Meta-analyses integrate the information from multiple studies and are suggested to be a powerful approach in detecting highly relevant and commonly affected pathways.

**Data sources:**

ArrayExpress, Gene Expression Omnibus and PubMed databases were screened for microarray gene expression profiling studies of MS and its experimental animal models.

**Study eligibility criteria:**

Studies comparing central nervous system (CNS) samples of diseased versus healthy individuals with n >1 per group and publically available raw data were selected.

**Material and Methods:**

Included conditions for re-analysis of differentially expressed genes (DEGs) were MS, myelin oligodendrocyte glycoprotein-induced experimental autoimmune encephalomyelitis (EAE) in rats, proteolipid protein-induced EAE in mice, Theiler’s murine encephalomyelitis virus-induced demyelinating disease (TMEV-IDD), and a transgenic tumor necrosis factor-overexpressing mouse model (TNFtg). Since solely a single MS raw data set fulfilled the inclusion criteria, a merged list containing the DEGs from two MS-studies was additionally included. Cross-study analysis was performed employing list comparisons of DEGs and alternatively Gene Set Enrichment Analysis (GSEA).

**Results:**

The intersection of DEGs in MS, EAE, TMEV-IDD, and TNFtg contained 12 genes related to macrophage functions. The intersection of EAE, TMEV-IDD and TNFtg comprised 40 DEGs, functionally related to positive regulation of immune response. Over and above, GSEA identified substantially more differentially regulated pathways including coagulation and JAK/STAT-signaling.

**Conclusion:**

A meta-analysis based on a simple comparison of DEGs is over-conservative. In contrast, the more experimental GSEA approach identified both, *a priori* anticipated as well as promising new candidate pathways.

## Introduction

As shown in various microarray studies of multiple sclerosis (MS) and its experimental models, gene expression profiling represents a potent and hypothesis-free method to analyze the complex pathogenesis of demyelination [1]–[9]. However, these transcriptomic studies use diverse methodologies, focus on different pathomechanisms and commonly display a low overlap of differentially expressed genes (DEGs). Meta-analyses are more powerful in detecting true qualitative effects and avoid rare and heterogeneous, thus less reliable outcomes [10]–[13]. Furthermore, meta-analyses efficiently tackle the publication bias which shifts the focus of single microarray studies on individual high scoring pathways [11].

Histologically, MS lesions are characterized by a variable degree of demyelination, remyelination, inflammation, gliosis, and axonal injury [14], [15]. In fact, based on morphology, four different patterns have been described in actively demyelinating MS lesions, indicating different pathomechanisms and a heterogenous etiology of the disease [16]. While T cell and macrophage infiltration is present in all of these patterns, a marked loss of oligodendrocytes is observed only in pattern III and IV. The discriminating feature of pattern I and II is prominent deposition of immunoglobulins and complement, which are found solely in pattern II. Although oligodendrocyte death is observed in both patterns III and IV, apoptotic oligodendrocytes are exclusively observed in pattern III [16]. The suggested etiologic heterogeneity of MS is reflected by the abundance and diversity of experimental models of demyelination. These include immune-mediated, virus-induced, genetic, and toxic models [2], [17], [18]. Experimental autoimmune encephalomyelitis (EAE) represents a traditional immune-mediated MS model which is classically induced by encephalitogenic antigens. The susceptibility to EAE and the course of the disease varies among animal species, strain as well as the used antigen [19]. Myelin oligodendrocyte glycoprotein (MOG)-induced EAE exhibits a disease course which is dependent on the dose and the used species and strain [19], [20]. For instance, a low dose of MOG(1–125) induces a relapsing-remitting disease course in Dark Agouti rats with a temporary recovery between the active disease stages [1], [21]. Other EAE models include proteolipid protein (PLP) inoculation in mice, which similarly induces a relapsing-remitting disease course [19].

Infection of susceptible mouse strains with Theiler’s murine encephalomyelitis virus (TMEV) represents a well-established infectious animal model of MS. Inoculation of susceptible mice with low virulent strains of TMEV is followed by a characteristic biphasic disease course with an initial transitory polioencephalitis with predominant manifestation within the first week post infection, followed by a chronic progressive demyelinating leukomyelitis [3], [22]–[26]. Depending on the experimental setup and used assay, demyelination starts between two and six weeks after the infection, and reaches a plateau approximately three to five months after the infection [3], [23], [27], [28]. In addition, certain genetic modifications have shown to be associated with central nervous system (CNS) demyelination and are used as another model system. As such, tumor necrosis factor (TNF)-overexpressing mice develop a chronic progressive inflammatory demyelinating disease with oligodendrocyte apoptosis and microglial activation in the early stages, followed by demyelination and secondary axonal damage in late stages [2], [17]. Furthermore, a diversity of toxic models such as local ethidium bromide injection or systemic cuprizone feeding have been used to study demyelination and remyelination [18], [29]–[32].

The aims of the present study were 1.) to re-analyze publically available microarray data sets of MS and its animal models employing a consistent methodology, 2.) to compare the results across species, experimental models and platforms in order to detect highly conserved pathways that offer the broadest therapeutic potential, and 3.) to explore, if the transcriptional changes in the different animal models reflect the anticipated difference in the pathomechanisms.

## Methods

### Data Selection

**Figure 1:**
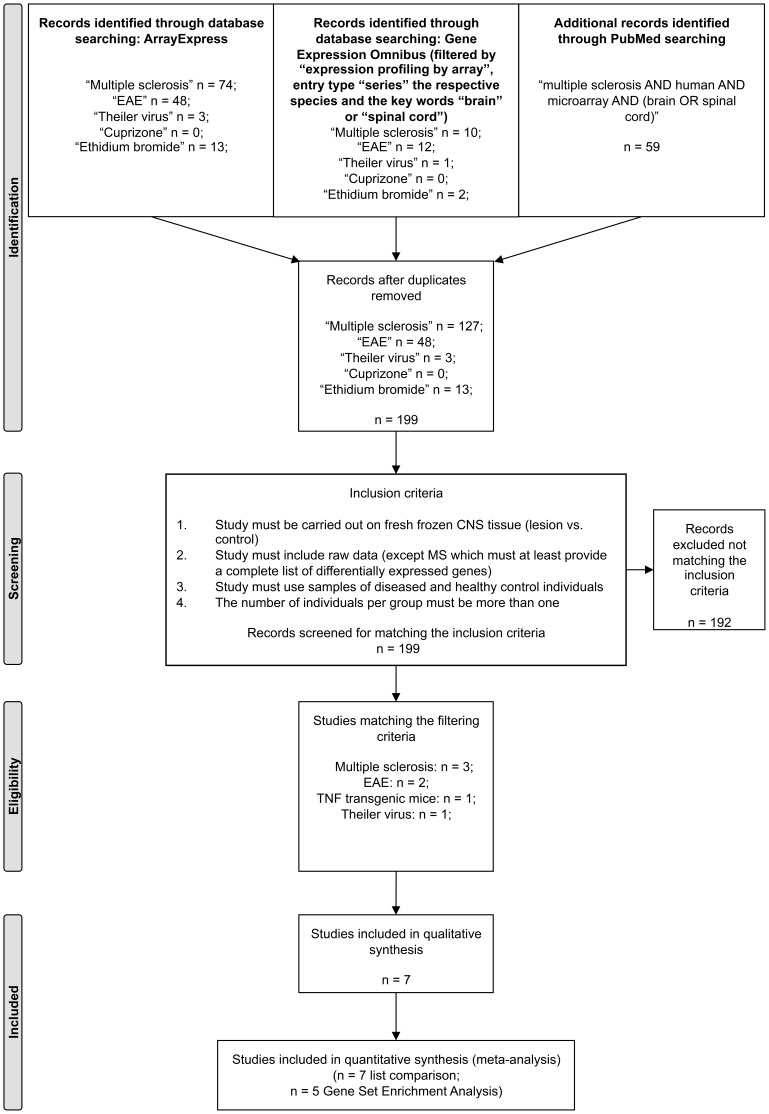
Flow diagram of systematic database search. ArrayExpress and Gene Expression Omnibus (GEO) were searched for microarray gene expression profiling studies with publically available raw data of multiple sclerosis and its experimental animal models with the search terms “Multiple sclerosis”, “EAE” and “Theiler virus”, “Cuprizone” and “Ethidium bromide”. For multiple sclerosis, additional information was gathered from PubMed database using the keywords “multiple sclerosis AND human AND microarray AND (brain OR spinal cord)”. All microarray studies published prior to October 24^th^ 2013, the last time-point of database search, were screened. n = number of records.

ArrayExpress and Gene Expression Omnibus (GEO) were searched for microarray gene expression profiling studies with publically available raw data of MS and its experimental animal models using the search terms “multiple sclerosis”, “EAE”, “Theiler virus”, “cuprizone” and “ethidium bromide” [33], [34]. For MS, additional information was gathered from the PubMed database using the keywords “multiple sclerosis AND human AND microarray AND (brain OR spinal cord)” (**Figure 1**) [35]. All recorded studies prior to October 24^th,^ 2013, the last time-point of the database quest, were screened. The inclusion criteria for the current meta-analysis were as follows: 1.) frozen CNS tissue is used, 2.) samples of diseased and healthy control individuals are compared, 3.) the number of diseased individuals per group is more than one, and 4.) complete raw data is publically accessible [13]. At the time of data collection only one MS microarray gene expression study fulfilled these inclusion criteria (GEO accession number: E-GEOD-38010) [9]. This study provides data of chronic-active MS plaques, chronic MS plaques, and control tissues of healthy individuals. Chronic active plaques were defined as chronic demyelinated lesions with sharply defined margins and recent areas of inflammatory demyelination at the edges. In contrast, chronic plaques showed demyelination with well demarcated borders and abundant astrogliosis, but lacked inflammatory cell infiltration [9]. As only this single MS study matched the inclusion criteria, we lowered the criteria for MS in order to broaden the data base. Therefore, we re-screened the literature for MS microarray studies that fulfill the aforementioned inclusion criteria 1–3, and at least provide a complete list of all differentially expressed genes (DEGs) with fold changes and p-values. Two additional studies of MS matched these lowered criteria. Both studies provide complete lists of DEGs from patients diagnosed with secondary progressive MS in comparison to healthy controls [5], [6]. Here, one study investigated both active and chronic active lesions [6], while the other study focused on active MS lesions [5]. In both studies, active lesions were defined as lesions with active demyelination as well as inflammatory infiltrates [5], [6].

Regarding the animal models, the original inclusion criteria were fulfilled by a study investigating three phases (acute, recovery and relapse) in MOG-induced EAE in Dark Agouti rats (ArrayExpress accession number: E-MEXP-1025) [1]. In this study, female Dark Agouti rats were immunized with recombinant MOG (amino acids 1–125), emulsified in complete Freund’s adjuvant. The acute phase of EAE was defined as the first EAE attack with clinical signs such as hind limb paralysis, whereas the recovery phase was defined as the first day at which the rats began to gain weight again. The relapsing phase presented as a second worsening of the clinical signs [1]. A second EAE study investigated a single time-point of acute clinical disease after PLP-inoculation in 8–12 weeks old SJL/J mice (GEO accession number: E-GEOD-44989) [8]. Furthermore, a time-series study, exploring day 14, 42, 98, and 196 post infection in TMEV-infected SJL/J mice was retrieved (ArrayExpress accession number: E-MEXP-1717) [3]. The mice exhibited a progressive inflammatory demyelinating leukomyelitis with progressively increasing locomotor deficits beginning on day 42 post infection [3]. Additionally, a study investigating the early and late phase of progressive demyelination in the transgenic TNF–overexpressing mouse strain Tg6074 fulfilled the criteria (ArrayExpress accession number: E-MTAB-1) [2]. While the early disease stage included mice up to three weeks of age the late stage comprised mice with an age of three to nine weeks.

A summary of all microarray studies that were finally included in the present meta-analysis is given in **Table 1**.

**Table 1:** Experimental setup of the individual gene expression microarray studies of multiple sclerosis, experimental autoimmune encephalomyelitis, Theiler’s murine encephalomyelitis virus-induced demyelinating disease and transgenic tumor necrosis factor-overexpressing mice included in the current meta-analysis.

Condition	Reference	ArrayExpressdatabaseaccession number	Platform	Probesets/cDNAs	Genes (UniGeneIds)	Species	Tissue	Experimental design
**Multiple sclerosis**	***Han et al. 2012***	E-GEOD-38010	Affymetrix HumanGenome U133Plus 2.0	54613	29035	Homo sapiens,male andfemale	brain	Secondary-progressive MS-patients (n = 4); 2 chronic-active and 2 chronic lesions; n = 2 healthy control samples
**Multiple sclerosis**	***Lindberg et al. 2004***	not applicable	Affymetrix HumanU95A Array	12627	9151	Homo sapiens,male andfemale	brain	Secondary progressive MS patients (n = 5); controls (n = 12)
**Multiple sclerosis**	***Tajouri et al. 2003***	not applicable	Custom Array	3965	3771	Homo sapiens,male andfemale	brain	Secondary-progressive MS-patients (n = 5); 2 acute and 3 chronic lesions; controls (n = 5)
**Experimental** **autoimmune** **encephalomyelitis**	***Mueller et al. 2008***	E-MEXP-1025	Affymetrix RatGenome U34Arrays A,B,C	26379	16010	Rattus norvegicus,Dark Agouti,female	spinalcord	Experimental study, one factorial design: 1.) control group (n = 3); 2.) EAE acute phase (n = 3); 3.) EAE recovery phase (n = 3); 4.) EAE relapsing phase (n = 4)
**Experimental** **autoimmune** **encephlalomyelitis**	www.ebi.ac.uk/arrayexpress/	E-GEOD-44989	Affymetrix MouseGenome U74Av2	12423	8900	Mus musculusSJL/J,female	spinalcord	Experimental study, one factorial design: 1) control group (n = 3); 2) diseased group (n = 5); pooled samples from two animals each were hybridized per array.
**Theiler’s murine encephalomyelitis virus-induced demyelinating disease**	***Ulrich et al. 2010***	E-MEXP-1717	Affymetrix MouseGenome 430 2.0 Array	45101	20447	Mus musculus, SJL/JHanHsd, female	spinalcord	Experimental study, two factorial design: Factor 1: Group (TMEV-infected, Mock-infected); Factor 2: days post infection (14 dpi, 42 dpi, 98 dpi, 196 dpi); independent groups with n = 6, except TMEV-infected 98 dpi, n = 5.
**Transgenic TNF-overexpressing mice (Tg6074)**	***Tseveleki et al. 2010***	E-MTAB-1	Custom A-TIGR-5Mouse27 k array, v.1	25581	13435	Mus musculus, TNF transgenic lineTg6074, male andfemale	brain	Experimental study, two factorial design: Factor 1: Group (Tg6074 [TNFtg], Wild type [WT]); Factor 2: Time (early: up to 3 weeks of age, late: 3 to 9 weeks of age). TNFtgearly (n = 6), TNFtglate (n = 5), WTearly (n = 2), WTlate (n = 3)

### Low Level Analysis

A complete re-analysis of the raw data was performed for one MS, both EAE, and the Theiler’s murine encephalomyelitis virus-induced demyelinating disease (TMEV-IDD) data sets, which are based on human, rat and mouse Affymetrix 3′IVT gene chips, respectively, employing Probe Logarithmic Intensity Error Estimation (PLIER +16) normalization. For the TIGR two color array based transgenic TNF–overexpressing mice (TNFtg) study, the publically available locally weighted scatterplot smoothing regression (LOWESS)-normalized data sets were used.

### Differentially Expressed Genes

P-values were calculated for each contrast using the linear models for microarray data (LIMMA)-method embedded in Babelomics 4.3 [36], [37]. To correct for multiple testing, q-values were calculated according to the method of Storey and Tibshirani using QVALUE 1.0. Filtering criteria for the selection of DEGs were a highly stringent fold change ≥2.0 or ≤ −2.0 and a moderately stringent q-value ≤0.05 in at least one of the pair-wise contrasts within the respective experiment. For the two additional MS studies without raw data, the lists of DEGs from the original studies were filtered employing a highly stringent fold change ≥2.0 or ≤ −2.0 and a moderately stringent p-value ≤0.05, respectively [38].

### Functional Annotation Clustering

Significantly overrepresented functional associations were selected from the biological process category of the gene ontology (GO) database using a modified Fisher’s Exact Test (EASE score) calculated in DAVID 6.7 [39], [40]. The resulting lists of significantly enriched GO terms were summarized into a manageable number of ≤10 enriched biological modules of functionally related GO terms employing the DAVID functional annotation clustering algorithm with customized settings. Enriched biological modules were ranked based on their respective enrichment score, which is calculated as the negative log_10_ of the geometric mean of the EASE scores of all comprised GO terms [39].

### Cross-study List Comparison

In order to compare the DEGs detected in MS, EAE, TMEV-IDD, and TNFtg across the different species, orthologous mouse UniGeneIDs were retrieved employing MADGene V5 [41]. Furthermore, to focus the present investigations on the demyelinating disease phases, only the data from the acute phase of MOG- as well as PLP-induced EAE, days 42, 98, and 196 post infection of TMEV-IDD, and the late stage of TNFtg were selected for comparative analysis. The lists of orthologous mouse UniGeneIDs were compared with regard to their intersections at the gene level using a Venn diagram (http://bioinfogp.cnb.csic.es/tools/venny/index.html.), followed by functional annotation clustering of the respective subsets as described above.

### Cross-study Gene Set Enrichment Analysis

The MS study of Han et al. (2012), MOG- and PLP-induced EAE, TMEV-IDD, and TNFtg were directly compared at the pathway level employing GSEA v2.0.10 [42]. Similar to the list comparison, only data from demyelinating disease phases were used for this comparative meta-analysis approach. Official human gene symbols (HUGO) required as input format in GSEA were retrieved employing MADGene V5 [41], [42]. The expression values of all 3788 genes (HUGO) present on all five array platforms were adjusted to a comparable scale by a division of each expression value by the mean expression of the controls within the respective experiment followed by log_2_-transformation (**Supplemental Table S1**). GSEA was used to identify commonly enriched GO terms of the biological process category in diseased (n = 35) versus control (n = 29) individuals, with a q value ≤0.1 [40], [42]. Subsequently, a leading edge analysis was performed in order to identify the genes that contribute most to the enrichment score of the enriched GO terms (leading edge genes) and the degree of overlap of these GO terms.

### Transcriptional Changes Reflecting MS Patterns I–IV

GO terms suggested to reflect the principal pathomechanistic features, which are used to discriminate patterns I–IV of MS by Lucchinetti et al. (2000) were selected using AmiGO version 1.8 [16], [40]. Consequently, a differential expression of the genes within the GO term “T cell mediated immunity” was expected to be found in all patterns I–IV. Pattern I was suggested to differ from Pattern II by a marked differential expression of genes of the GO term “immunoglobulin mediated immune response” in pattern II only. Marked differential expression of the genes belonging to the GO term “myelination” was anticipated to be present in patterns III and IV only. A differential expression of the genes comprised by the GO term “positive regulation of apoptotic process” is presumably indicative of an affiliation to pattern III, rather than pattern IV. Only genes represented on all array platforms were included in the statistical analysis comparing the frequency of DEGs between EAE, TMEV-IDD, and TNFtg within each gene signature using Cochran’s Q and McNemar *post-hoc* tests employing IBM SPSS Statistics.

## Results

### Differentially Expressed Genes in MS

The re-analysis of the study of Han et al. (2012) revealed a total number of 4686 genes (**Supplemental Table S2**) that were considered as differentially expressed in either chronic active or chronic MS plaques compared to healthy control individuals. 4583 DEGs were detected in chronic active plaques, whereas 3695 genes were differentially regulated in chronic plaques. Functional annotation clustering revealed multiple similar enriched biological modules in both plaque types (**Table 2**). The highest scoring biological module associated with up-regulated genes was related to axonogenesis, while the highest scoring biological module associated with down-regulated genes was related to myelination (**Table 2** **)**.

**Table 2:** Differentially expressed genes and enriched biological modules in multiple sclerosis as revealed by re-analysis of publically available microarray data and published gene lists [5], [6], [9].

Time	Probe sets		Genes	Enriched biological modules
**Multiple sclerosis,** **chronic-active** **plaques ** [**9**]	**up**	3486	2650	Axonogenesis (ES = 6.28), hormone transport (ES = 2.18), negative regulation of actin filament polymerization (ES = 1.52), regulation of cyclase activity (ES = 1.22)
	**down**	2910	1933	Myelination (ES = 2.01), axonogenesis (ES = 1.35)
**Multiple sclerosis,** **chronic plaques ** [**9**]	**up**	2865	2202	Axonogenesis (ES = 7.58), hormone transport (ES = 1.55), negative regulation of adenylate cyclase activity (ES = 1.45)
	**down**	2616	1493	Myelination (ES = 1.70), neural tube formation (ES = 1.50), positive regulation of transcription from RNA polymerase II promoter (ES = 1.36), axonogenesis (ES = 1.28)
**Multiple sclerosis, chronic** **active lesions ** [**5**]	**up**	94	84	Striated muscle cell development (ES = 1.54), peptide hormone secretion (ES = 1.46), regulation of adenylate cyclase activity (ES = 1.20), axonogenesis (ES = 0.66), regulation of membrane potential (ES = 0.61)
	**down**	35	34	Negative regulation of neuron apoptosis (ES = 1.40), positive regulation of MAP kinase activity (ES = 1.21), regulation of cell morphogenesis involved in differentiation (ES = 0.98), system development (ES = 0.79), cellular ion homeostasis (ES = 0.44)
**Multiple sclerosis,** **acute lesions ** [**6**]	**up**	44	44	Cellular carbohydrate catabolic process (ES = 1.02), myelination (ES = 1.94), purine nucleotide biosynthetic process (ES = 1.46), response to steroid hormone stimulus (ES = 1.40), positive regulation of apoptosis (ES = 1.34)
	**down**	43	43	DNA repair (ES = 0.98), protein catabolic process (ES = 0.61), positive regulation of cellular metabolic process (ES = 0.48), regulation of cellular metabolic process (ES = 0.16), macromolecule biosynthetic process (ES = 0.12)
**Multiple sclerosis,** **chronic active lesions ** [**6**]	**up**	38	38	Cellular homeostasis (ES = 5,36), ATP biosynthetic process (ES = 2.20), myelination (ES = 2.04), positive regulation of macromolecule biosynthetic process (ES = 0.87)
	**down**	42	42	Receptor-mediated endocytosis (ES = 0.93), germ cell development (ES = 0.72), modification-dependent protein catabolic process (ES = 0.26)

ES = enrichment score.

The merged list of DEGs from the two additionally included MS studies comprised 232 human genes (**Supplemental Table S3**). Notably, the DEGs of acute lesions and chronic active lesions within the study of Tajouri at al. (2003) showed a high overlap, whereas the DEGs of chronic active lesions of Tajouri et al., 2003 compared to the chronic active lesions of the study of Lindberg et al. (2004) displayed a comparatively low overlap (**Table 2**). On the functional level an up-regulation of genes associated with the terms axonogenesis and regulation of adenylate cylase activity was a prominent feature of chronic-active plaques in the study of Lindberg et al. (2004) as well as in chronic plaques of the study by Han et al. (2012). Enriched GO terms related to myelination were retrieved for the down-regulated genes in both plaque types investigated by Han et al. (2012) as well as in the up-regulated genes of both investigated plaque types of Tajouri et al. (2003) (**Table 2**). The intersection of the DEGs of all three independent studies only contained the two genes e*phrin receptor B6 (EPHB6)* and *glial cell derived neurotrophic factor family receptor alpha 2 (GFRA2)*.

### Differentially Expressed Genes in EAE

In MOG-induced EAE in Dark Agouti rats, a total of 2957 rat genes (**Supplemental Table S4**) were considered as differentially expressed in one or more of the three disease phases. In the acute and recovery phase a comparable amount of 1633 and 1746 DEGs was detected, respectively (**Table 3**). An explicitly lower number of 432 DEGs was detected in the relapse phase of EAE. Functional annotation clustering revealed three enriched biological modules of up-regulated genes associated with “positive regulation of adaptive immune response”, “adaptive immune response”, and “positive regulation of type II hypersensitivity” in all three phases of the disease.

**Table 3:** Differentially expressed genes and enriched biological modules in experimental autoimmune encephalomyelitis as revealed by re-analysis of publically available microarray data [1], [8].

Time	Probe sets		Genes	Enriched biological modules
**MOG-induced experimental autoimmune encephalomyelitis, acute phase ** [**1**]	**up**	1213	897	Adaptive immune response (ES = 12.58), positive regulation of lymphocyte activation (ES = 9.00), positive regulation of adaptive immune response (ES = 5.03), cell migration (ES = 4.95), positive regulation of innate immune response (ES = 4.62), response to host (ES = 2.62), positive regulation of type II hypersensitivity (ES = 2.56)
	**down**	1010	736	Regulation of neuron differentiation (ES = 3.85), cell morphogenesis involved in neuron differentiation (ES = 3.43), negative regulation of microtubule depolymerization (ES = 2.73)
**MOG-induced experimental autoimmune encephalomyelitis, recovery phase ** [**1**]	**up**	1080	836	Immunoglobulin mediated immune response (ES = 6.60), positive regulation of adaptive immune response (ES = 4.33), positive regulation of lymphocyte proliferation (ES = 2.86), positive regulation of leukocyte activation (ES = 2.79), regulation of type II hypersensitivity (ES = 2.79), positive regulation of phosphorylation (ES = 2.64), neuron projection morphogenesis (ES = 2.17)
	**down**	1213	910	Acetyl-CoA catabolic process (ES = 4.63), glycolysis (ES = 4.35), ATP biosynthetic process (ES = 1.96), regulation of microtubule depolymerization (ES = 1.96), nucleoside biosynthetic process (ES = 1.48)
**MOG-induced experimental autoimmune encephalomyelitis, relapse phase ** [**1**]	**up**	331	244	Antigen processing and presentation of peptide antigen via MHC class II (ES = 14.29), immunoglobulin mediated immune response (ES = 9.57), regulation of T cell proliferation (ES = 5.92), positive regulation of adaptive immune response (ES = 4.65), positive regulation of type II hypersensitivity (ES = 3.89), humoral immune response (ES = 3.42), induction of apoptosis (ES = 3.22)
	**down**	264	188	Cholesterol biosynthetic process (ES = 6.53)
**PLP-induced experimental autoimmune encephalomyelitis** [**8**]	**up**	502	434	B cell mediated immunity (ES = 12.97), chemotaxis (ES = 9.38), positive regulation of lymphocyte mediated immunity (ES = 6.53), positive regulation of lymphocyte activation (ES = 6.32), positive regulation of T cell mediated immunity (ES = 5.56), complement activation (ES = 4.74), positive regulation of T cell activation (ES = 4.08), T cell selection (ES = 2.19)
	**down**	41	38	nervous system development (ES = 3.21), cellular homeostasis (ES = 3.20)

ES = enrichment score.

In PLP-induced EAE in SJL/J mice a total of 472 mouse genes (**Supplemental Table S5**) were considered as differentially expressed in the surveyed acute stage. Functional annotation clustering revealed several enriched GO terms associated with T and B cell reponses, including the term “positive regulation of lymphocyte activation”, which was also retrieved for the acute disease stage of MOG-induced EAE (**Table 3**).

A comparison of PLP-induced EAE versus MOG-induced EAE resulted in an overlap of 254 DEGs in both EAE models.

### Differentially Expressed Genes in TMEV-IDD

A total of 679 mouse genes (**Supplemental Table S6**) were considered as differentially expressed at one or more of the four time-points. A comparison of the DEGs at the different time points revealed a minor amount of 138 up-regulated genes at 14 days post infection (dpi; **Table 4**). Higher numbers of DEGs were detected in the chronic demyelinating phase of the disease with a peak of 583 DEGs at 98 dpi**.** The vast majority of the DEGs in TMEV-IDD were up-regulated. Functional annotation clustering revealed enriched biological modules of up-regulated genes associated with “immunoglobulin mediated immune response”, “classical pathway of complement activation”, and “induction of apoptosis” at all four time points.

**Table 4:** Differentially expressed genes and enriched biological modules in Theiler’s murine encephalomyelitis virus-induced demyelinating disease, as revealed by re-analysis of publically available microarray data [3].

Time	Probe sets		Genes	Enriched biological modules
**Theiler’s murine** **encephalomyelitis** **virus-induced** **demyelinating** **disease, 14 dpi**	**up**	181	138	Positive regulation of immune response (ES = 13.64), immunoglobulin mediated immune response (ES = 8.83), positive regulation of adaptive immune response (ES = 5.97), positive regulation of T cell activation (ES = 5.77), antigen receptor-mediated signaling pathway (ES = 4.82), complement activation, classical pathway (ES = 3.65), positive regulation of T cell mediated cytotoxicity (ES = 3.52), induction of apoptosis (ES = 3.02), positive thymic T cell selection (ES = 2.90)
	**down**	0	0	n.s.
**Theiler’s murine** **encephalomyelitis** **virus-induced** **demyelinating** **disease, 42 dpi**	**up**	614	455	Immunoglobulin mediated immune response (ES = 18.38), complement activation, classical pathway (ES = 7.50), chemotaxis (ES = 6.69), induction of apoptosis (ES = 6.38), positive regulation of type II hypersensitivity (ES = 5.54), negative regulation of mononuclear cell proliferation (ES = 4.09), negative regulation of lymphocyte mediated immunity (ES = 3.64), positive regulation of isotype switching to IgG isotypes (ES = 2.70), positive regulation of interleukin-1 beta secretion (ES = 2.13)
	**down**	0	0	n.s.
**Theiler’s murine** **encephalomyelitis** **virus-induced** **demyelinating disease,** **98 dpi**	**up**	794	581	Positive regulation of immune response (ES = 31.78), immunoglobulin mediated immune response (ES = 16.87), chemotaxis (ES = 7.91), induction of apoptosis (ES = 7.05), positive regulation of phagocytosis (ES = 6.31), complement activation, classical pathway (ES = 5.85), positive regulation of type II hypersensitivity (ES = 5.02), negative regulation of mononuclear cell proliferation (ES = 4.18), positive regulation of isotype switching to IgG isotypes (ES = 3.98)
	**down**	2	2	n.s.
**Theiler’s murine** **encephalomyelitis** **virus-induced** **demyelinating disease,** **196 dpi**	**up**	785	579	Immunoglobulin mediated immune response (ES = 19.38), induction of apoptosis (ES = 7.39), positive regulation of phagocytosis (ES = 5.75), complement activation, classical pathway (ES = 5.36), positive regulation of T cell mediated cytotoxicity (ES = 5.18), positive regulation of type II hypersensitivity (ES = 5.17), negative regulation of mononuclear cell proliferation (ES = 3.35)
	**down**	3	2	n.s.

ES = Enrichment score; dpi = days post infection; n.s. = no significantly enriched gene ontology terms.

### Differentially Expressed Genes in TNFtg

A total of 173 mouse genes (**Supplemental Table S7**) were considered as differentially expressed in at least one of the two disease phases. An explicitly higher number of 162 DEGs was detected in the late phase of the disease, whereas only 11 DEGs were observed in the early phase (**Table 5**). The vast majority of DEGs in TNFtg were up-regulated. Functional annotation clustering revealed enriched biological modules only for the up-regulated genes in the late phase of the disease, and the highest scoring biological module was related to the classical pathway of complement activation.

**Table 5:** Differentially expressed genes and enriched biological modules in transgenic tumor necrosis factor-overexpressing mice as revealed by re-analysis of publically available microarray data [2].

Time	Probe sets		Genes	Enriched biological modules
**Transgenic TNF- overexpressing mice, early phase**	**up**	12	7	n.s.
	**down**	5	4	n.s.
**Transgenic TNF- overexpressing mice, late phase**	**up**	211	162	Complement activation, classical pathway (ES = 1.93), negative regulation of transcription factor activity (ES = 1.10), blood circulation (ES = 1.01), regulation of protein polymerization (ES = 0.91), cellular ion homeostasis (ES = 0.89)
	**down**	2	0	n.s.

ES = Enrichment score; n.s. = no significantly enriched gene ontology terms.

### Cross-study List Comparison

**Figure 2:**
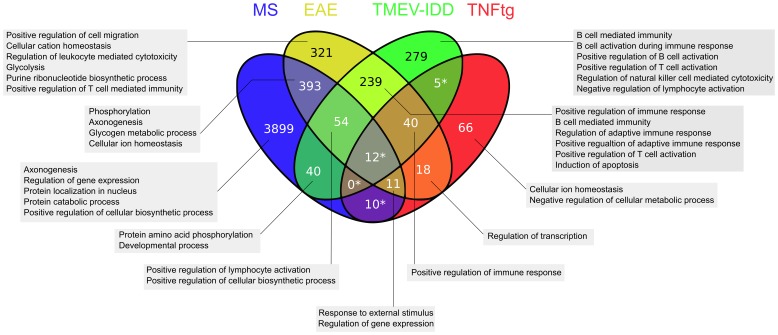
Venn diagram of the list comparison of differentially expressed genes (DEGs). Intersections comparing lists of DEGs in multiple sclerosis (MS), experimental autoimmune encephalomyelitis (EAE), Theiler’s murine encephalomyelitis virus-induced demyelinating disease (TMEV-IDD) and transgenic tumor necrosis factor-overexpressing mice (TNFtg). The numbers in the subsets represent the numbers of the comprised differentially expressed genes, and the grey boxes list the associated enriched biological modules. * = subsets without associated significantly enriched biological modules.

A comparison of the lists of 4423, 1388, 673 and 162 orthologous mouse UniGeneIDs corresponding to the DEGs detected in the demyelinating stages of MS, EAE, TMEV-IDD, and TNFtg, respectively, retrieved only 12 DEGs that were common in MS and the investigated animal models (**Figure 2**). Functional annotation clustering of these genes failed to detect significantly enriched biological modules. However, manual analysis of the genes revealed a predominant association to macrophage response and lysosomal degradation (**Table 6**). Interestingly, the majority of these genes was up-regulated in the animal models, while in MS the entire 12 genes were down-regulated (**Table 6**). 40 genes were found to be commonly differentially expressed in EAE, TMEV-IDD, and TNFtg (**Supplemental Table S8**). Functional annotation clustering of these genes revealed enrichment of the biological module “positive regulation of immune response”.

**Table 6:** Common differentially expressed orthologous mouse genes in Theiler’s murine encephalomyelitis virus-induced demyelinating disease (TMEV-IDD), myelin oligodendrocyte glycoprotein (MOG)- and proteolipid protein (PLP)-induced experimental autoimmune encephalomyelitis (EAE), tumor necrosis factor-overexpressing mice (TNFtg), and multiple sclerosis (MS).

UniGeneID	Genesymbol	Genetitle	TMEV-IDD			MOG-EAE	PLP-EAE	TNFtg	MS	
			FCday 42	FCday 98	FCday 196	FC	FC	FC	FC chronic active	FC chronic
Mm.101034	Tgif1	TG interacting factor 1	1.61*	1.96*	2.31*	8.46*	3.22*	2.57*	−2.98*	−3.18*
Mm.171378	Ucp2	Uncoupling protein 2(mitochondrial, proton carrier)	1.50*	2.15*	2.05*	3.56*	−1.03	3.99*	−3.34*	−4.19*
Mm.219527	C1s	Complement component 1, s subcomponent	2.44*	2.97*	3.07*	2.84*	n.a.	6.65*	−6.11*	−6.02*
Mm.271868	Laptm5	Lysosomal-associated protein transmembrane 5	2.91*	3.91*	3.09*	6.45*	3.97*	4.59*	−15.28*	−9.27*
Mm.3317	Gusb	Glucuronidase, beta	2.07*	2.93*	3.22*	2.37*	3.12*	2.92*	−2.42*	−2.32*
Mm.14455	Tgfbi	Transforming growth factor,beta induced	2.20*	2.32*	2.16*	n.a.	17.83*	8.21*	−4.43*	−3.74
Mm.15819	Cd68	CD68 antigen	5.26*	9.54*	8.93*	19.50*	6.30*	6.91*	−2.15*	−1.95
Mm.22574	Csf1r	Colony stimulating factor1 receptor	2.08*	2.09*	1.70*	4.66*	3.03*	2.71*	−3.66*	−3.99*
Mm.2277	Ctsh	Cathepsin H	3.21*	4.93*	4.50*	2.40*	6.81*	5.49*	−3.49*	−3.53*
Mm.30010	Arpc1b	Actin related protein 2/3 complex, subunit 1B	1.88*	2.83*	2.95*	6.22*	4.09*	3.63*	−2.13*	−1.89*
Mm.3152	Lgals3bp	Lectin, galactoside-binding,soluble, 3 binding protein	8.88*	10.61*	7.43*	6.13*	6.24*	23.31*	−11.15*	−10.28*
Mm.4219	Man2b1	Mannosidase 2, alpha B1	1.79*	2.52*	2.65*	2.45*	3.35*	2.71*	−2.38*	−2.48*

FC = Fold change;

* significant different gene expression as compared to controls (q <0.05); n.a. = not analyzed (gene not represented on array).

### Cross-study Gene Set Enrichment Analysis

**Figure 3:**
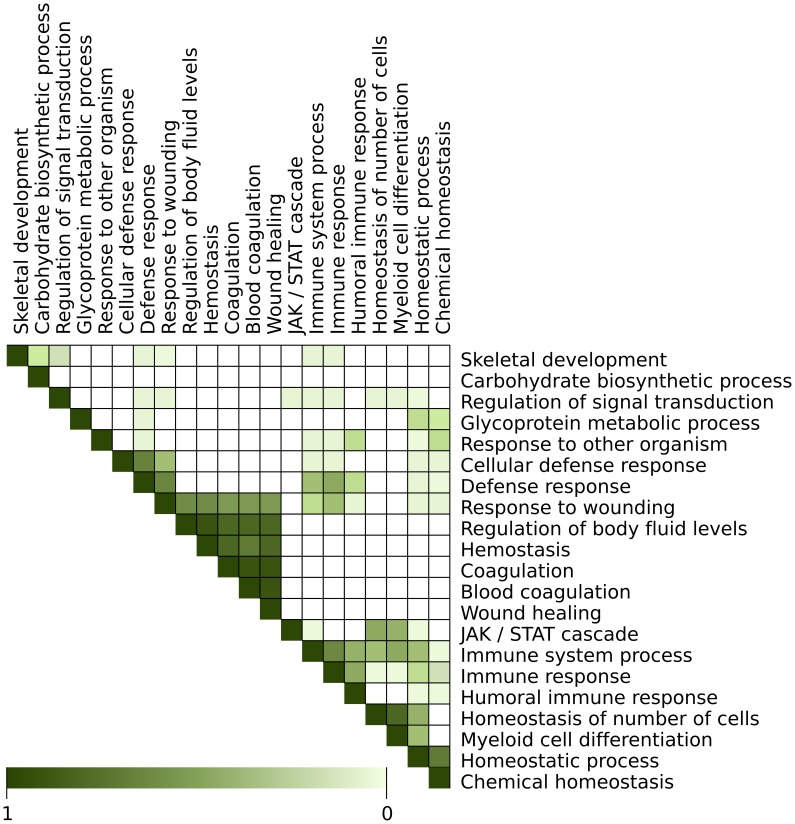
Gene set similarity map retrieved by Gene Set Enrichment Analysis (GSEA). Pathways significantly enriched and positively correlated with disease versus control in multiple sclerosis, experimental autoimmune encephalomyelitis, Theiler’s murine encephalomyelitis virus-induced demyelinating disease and transgenic tumor necrosis factor-overexpressing mice as revealed by cross-study Gene Set Enrichment Analysis. The graph displays a similarity map of GO terms, retrieved by the leading edge analysis GSEA. The intensity of the colour gradient represents a measure for the relative overlap of genes in the respective GO terms, ranging from 100% overlap (dark green) to 0% overlap (white) of the leading edge genes within the GO terms on the x- and y- axis.

GSEA was used as an alternative pathway-centered approach to identify commonly affected gene sets in the demyelinating stages of MS, EAE, TMEV-IDD, and TNFtg. Accordingly, 21 GO terms of the biological process category were identified as being positively correlated with diseased versus control individuals in the merged data set (**Table 7**). The subsequent leading edge analysis pointed out that some of these GO terms displayed a major overlap of the comprised genes (**Figure 3** **; Supplemental Table S9**). Therefore, the affected GO terms could be manually summarized into seven biological modules such as “immune response” (four GO terms), “defense response” (four GO terms), “coagulation” (six GO terms), “homeostasis” (four GO terms), “regulation of signal transduction”, “carbohydrate biosynthetic process”, and “skeletal development”.

**Table 7:** Analysis of commonly affected gene sets (gene ontology terms) in diseased versus control individuals in multiple sclerosis, experimental autoimmune encephalomyelitis, Theiler’s murine encephalomyelitis virus-induced demyelinating disease, and transgenic tumor necrosis factor-overexpressing mice on the pathway level employing Gene Set Enrichment Analysis.

Gene Set	Genes (N)	Normalizedenrichment score	p-value	q-value
Immune system process	80	2.060	<0.001	0.039
Cellular defense response	13	1.892	<0.001	0.041
Homeostatic process	50	1.934	<0.001	0.043
Homeostasis of number of cells	11	1.937	<0.001	0.047
Immune response	52	1.985	<0.001	0.048
Defense response	48	1.857	<0.001	0.058
JAK/STAT cascade	11	1.818	<0.001	0.062
Humoral immune response	10	1.703	<0.001	0.076
Coagulation	15	1.705	0.023	0.079
Myeloid cell differentiation	14	1.694	0.020	0.079
Blood coagulation	15	1.705	0.023	0.083
Regulation of body fluid levels	16	1.709	0.023	0.084
Response to wounding	46	1.763	<0.001	0.088
Hemostasis	16	1.709	0.023	0.090
Regulation of signal transduction	68	1.771	<0.001	0.091
Skeletal development	28	1.671	0.023	0.094
Response to other organism	15	1.721	<0.001	0.095
Glycoprotein metabolic process	34	1.709	<0.001	0.095
Wound healing	16	1.649	<0.001	0.097
Carbohydrate biosynthetic process	16	1.652	<0.001	0.100
Chemical homeostasis	30	1.643	<0.001	0.100

N = number of genes in the gene set.

### Transcriptional Changes Reflecting MS Patterns I–IV

**Figure 4:**
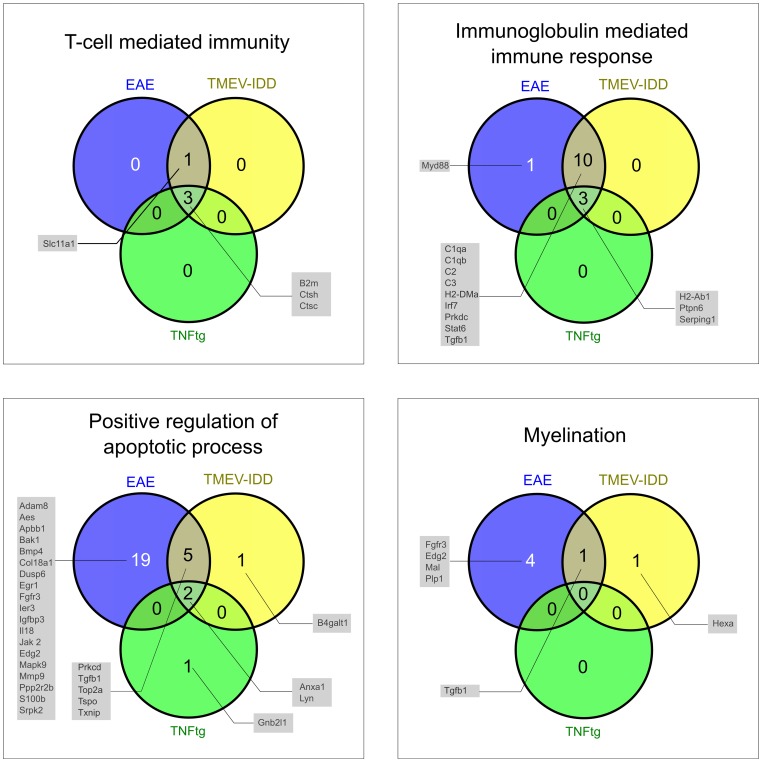
Venn diagram of differentially expressed genes within each gene signature. Intersections of differentially expressed genes in experimental autoimmune encephalomyelitis (EAE), Theiler’s murine encephalomyelitis virus-induced demyelinating disease (TMEV-IDD) and transgenic tumor necrosis factor-overexpressing mice (TNFtg) within the gene signatures for the GO terms “T cell mediated immunity”, “immunoglobulin mediated immune response”, “positive regulation of apoptotic process” and “myelination”. The numbers in the intersections represent the absolute numbers of the comprised differentially expressed genes, and the grey boxes list the associated gene symbols.

The gene signatures for “T cell mediated immunity”, “immunoglobulin mediated immune response”, “positive regulation of apoptosis”, and “myelination” comprised 13, 23, 123, and 29 mouse UniGeneIDs present on all four array platforms, respectively (**Supplemental Table S10**). 23–30% of the genes comprised by the GO term “T cell mediated immunity” were observed to be differentially expressed in EAE, TMEV-IDD, and TNFtg with a relatively high overlap of the DEGs between the studies. No significant different percentage of DEGs was detectable between the three animal models (Cochran’s Q: p = 0.368). Three DEGs, *beta-2-microglobulin* (*B2m*), *cathepsin H* (*Ctsh*), and *cathepsin C* (*Ctsc*) were found to be up-regulated in all three animal studies (**Figure 4**).

Concerning the GO term “immunoglobulin mediated immune response”, a significantly higher percentage of DEGs was detected in EAE (60%) and TMEV-IDD (56%) as compared to 13% DEGs in TNFtg (Cochran’s Q: p<0.001) (**Figure 4**). The intersection of all three animal models contained the up-regulated genes *histocompatibility 2, class II antigen A, beta 1* (*H2-Ab1*), *protein tyrosine phosphatase, non-receptor type 6* (*Ptpn6*), and *serine (or cysteine) peptidase inhibitor, clade G, member 1* (*Serping1*).

None of the genes comprised by the GO term “myelination” were differentially expressed in TNFtg. In contrast, 7% and 17% DEGs involved in myelination were observed for TMEV-IDD and EAE, respectively. However, statistical analysis did not reach the level of significance for this GO term (Cochran’s Q: p = 0.091).

Regarding the GO term “positive regulation of apoptotic process” EAE displayed the highest relative amount with 21% DEGs resulting in a significant difference compared to both TMEV-IDD (7% DEGs) and TNFtg (2% DEGs) (Cochran’s Q: p<0.001) (**Figure 4**). The intersection of all animal models contained the up-regulated genes *annexin A1* (*Anxa1*) and *Yamaguchi sarcoma viral (v-yes-1) oncogene homolog* (*Lyn*).

## Discussion

The present re-analysis of publically available data sets of MS, EAE, TMEV-IDD, and TNFtg generally displayed a lower number of DEGs as compared to the original studies. This might be attributed to the highly stringent filtering criteria suggested to be essential for high reproducibility across studies and platforms [38]. However, at the level of the most severely affected biological modules, the current results are in general agreement with the original studies [1]–[3], [5], [6], [8], [9].

Meta-analyses of microarray experiments can be broadly divided into analyses combining statistical significance across studies 1.) on the gene level, including list comparisons, and 2.) on the pathway level, including cross-study GSEA [10], [12]. In the present analysis, the list comparison method did only retrieve 12 DEGs that were commonly affected in MS, EAE, TMEV-IDD, and TNFtg, supporting previous studies suggesting that list comparisons are an over-conservative approach [10]. Notably, 8 out of these 12 common DEGs are known to be expressed by macrophages [43]–[52]. Interestingly, all of the 12 DEGs in the intersection were down-regulated in MS, while they were up-regulated in the animal models. The reason for this dis-concordance remains unclear. However, this observation is in agreement with the recently reported poor transcriptional overlap of mouse models and human inflammatory diseases [53]. Furthermore, the complex role of macrophages in the pathogenesis of MS is still not understood in detail [54]. *Lysosomal-associated protein transmembrane 5* is a potent regulator of pro-inflammatory signaling pathways in macrophages, *transforming growth factor interacting factor 1* is a regulator of murine macrophage activation, *transforming growth factor beta, induced*, is up-regulated in macrophages following phagocytosis of apoptotic cells, *colony stimulating factor 1 receptor 1* signaling is known to be involved in macrophage proliferation and migration, and *cathepsin H* have been found to be expressed by activated microglia and macrophages, CD68 represents a well-known marker for macrophages and activated microglia, *uncoupling protein 2* has been described to be differentially regulated following lipopolysaccharide stimulation of macrophages, and *complement component 1, s subcomponent*, is constitutively expressed in bone-marrow derived macrophages [43]–[52]. *Uncoupling protein 2* (*Ucp2*) is additionally a neuroprotector and neuromodulator in the central nervous system as it may decrease oxidant damage due to regulation of the production of reactive oxygen species such as superoxide [55], [56]. In agreement to our findings, *Ucp2* is known to be differentially expressed in both EAE and MS [56], [57]. Interestingly, alterations in this protein are a risk factor for MS [58], highlighting this gene as a hub gene involved in the pathogenesis of demyelinating diseases.

The 40 DEGs commonly affected in EAE, TMEV-IDD, and TNFtg, as well as the mutual 239 DEGs in EAE and TMEV-IDD were related to biological modules associated with an activation of the adaptive immune response thus reflecting the anticipated changes in these animal models [1], [3], [7]. The limited concordance of DEGs in the list comparisons of MS may be at least partially attributed to the marked genetic complexity and interspecies diversity, which hampers the detection of orthologous genes [4], [59]. An example for this dilemma is the lack of orthology. One example for this problem are the DEGs coding for the immunoglobulin kappa light chain: MS (*immunoglobulin kappa constant*), EAE (*immunoglobulin kappa chain variable* (*Igkv*) *28*), and TMEV-IDD (*Igk-V1, Igkv14–111; Igkv15–103, Igkv19–93, Igk-V28, Igkv4–55, Igkv4–68, Igkv4–72, Igkv6–14, Igkv6–20, Igkv8–30*). Although these DEGs all code for parts of the same molecule, the complex species-specific conformation and nomenclature of the immunoglobulin light chain gene cluster prevented automatic detection of this analogous change in gene expression.

A possible alternative to circumvent the problems of orthologous gene assignments in cross-species comparisons is a shift from the gene level to the more unifom pathway level [60]. This was done by a cross-study GSEA evaluating MS, EAE, TMEV-IDD and TNFtg for commonly affected pathways. Accordingly, in addition to the already anticipated immune response pathways, GSEA highlighted the common biological module “coagulation” in MS, EAE, TMEV-IDD and TNFtg. The observation of a cluster of 6 GO terms comprising 47 genes involved in coagulation and hemostasis supports the results of a proteomic analysis of MS [61]. Concordantly, the thrombin inhibitor hirudin leads to a dramatic improvement in disease severity in EAE [61]. Furthermore, fibrinogen depletion leads to an increased lifespan, retardation of the clinical symptoms and delayed inflammation and demyelination in TNFtg [62]. The importance of the coagulation cascade in disease development has also been shown in a treatment study with batroxobin, a thrombin-like defibrinogenating enzyme, in TMEV-IDD, which resulted in decreased clinical signs and reduced CNS demyelination in treated animals [63].

Based on the detailed histological and immunohistological descriptions of Lucchinetti et al. (2000) [16], we assumed that demyelinating conditions analogous to all four MS patterns should be associated by a transcriptional up-regulation of genes comprised by the GO term “T cell mediated immunity”. Accordingly, a moderate percentage of DEGs associated with this gene signature was detected in EAE, TMEV-IDD, and TNFtg. This transcriptional change reflects the histological demonstration of inflammatory T cells and macrophages within the lesions in MOG-induced EAE, TMEV-IDD, and TNFtg as shown in previous studies [3], [17], [64]. Conditions analogous to MS pattern II were anticipated to be accompanied by an additional up-regulation of genes comprised by the GO term “immunoglobulin mediated immune response”. This GO term comprises genes involved in the synthesis of immunoglobulins as well as complement factors and therefore includes both important features indicative of MS pattern II. Accordingly, we observed a high percentage of DEGs associated to this gene signature in EAE and TMEV-IDD, and a significantly lower percentage in TNFtg. This result supports the hypothesis that a type II autoimmunity analogous to pattern II of MS is an important pathogenic feature of TMEV-IDD and certain subtypes of EAE [1], [3], [64], [65]. Furthermore, this reflects the histological demonstration of immunoglobulin, complement, B cells and plasma cells within the lesions of MOG-induced EAE and TMEV-IDD [3], [64], [65]. The pathogenic role of immunoglobulins in EAE remains controversial. However, several types of EAE in mice, rats, and monkeys are accompanied by B cell responses and immunoglobulin deposition [66]. In detail, deposition of immunoglobulin has been described in MOG(1–125)-induced EAE in Dark Agouti rats, one of the models used in the present meta-analysis [64]. However, studies in B cell deficient mice have shown that B cells are not critical for the development of MOG-induced murine EAE [67].

Experimental models with a marked oligodendrocyte dystrophy such as cuprizone-induced toxicity analogous to the MS patterns III and IV are characterized by an early-onset and marked down-regulation of genes involved in myelination [68]. Notably, none of the experimental models included in this study displayed a high percentage of DEGs included in the GO term “myelination”, suggesting that neither EAE, TMEV-IDD nor TNFtg display transcriptional changes anticipated to dominate in oligodendrocyte dystrophy analogous to patterns III and IV of MS. The majority of the DEGs from the myelination gene signature displayed a down-regulation in EAE. This is in agreement with the demonstrated down-regulation of multiple classical myelin genes in the range of a ∼−2- to −4-fold change comparing acute EAE with controls in the original publication of the MOG-induced EAE data set [1]. The magnitude of the transcriptional change roughly parallels the degree of demyelination, which is estimated to affect slightly more than one half of the spinal cord in MOG-induced EAE in female Dark Agouti rats [64]. Similarly, in TMEV-IDD a down-regulation of PLP and MBP mRNA to approximately 58% of control levels has been described using in-situ hybridization [69], and a down-regulation of MBP mRNA to approximately 70% of control levels has been described using RT-qPCR [23].

Based on the abundant apoptotic oligodendroglial cell death in pattern III, and non-apoptotic oligodendroglial cell death in pattern IV a respective differential expression of the genes of the GO term “positive regulation of apoptotic process” was suggested to differentiate between analogous conditions [16]. Although the TNFtg model is reported to display primary oligodendrocyte apoptosis with subsequent myelin loss as the predominant pathological feature [17], [62], we were unable to detect corresponding transcriptional changes in the genes comprised by the selected GO terms. The reason for this lack of concordance remains unclear. The only GO term observed in our re-analysis of this model which is possibly linked to programmed cell death was “ion homeostasis”. It is known that TNF potently increases intracellular Ca^2+^ and may thereby induce apoptosis [70]. Similar to our results, the original study describing the TNFtg data set also lacks the description of transcriptional changes associated with myelination and apoptosis [2]. It cannot be excluded that previous studies may have overestimated the relative contribution of oligodendrocyte apoptosis in comparison to the pro-inflammatory effects of TNF-overexpression in the pathogenesis of the TNFtg model. Alternatively, it seems plausible that the induction of apoptosis in oligodendrocytes via the p55TNF receptor-signaling pathway may simply occur by activation of the preformed apoptotic cascade without any related transcriptional changes. Further studies are needed to unravel, whether alternate gene signatures are better suited or if transcriptional profiling is generally a poor approach to reflect the degree of demyelination and apoptosis. The up-regulation of apoptosis–related genes in EAE is suggested to be linked to the marked infiltration of T cells, which are known for their marked expression of extrinsic apoptosis pathway genes like Fas and Fas-L [71]. Apoptosis is known to mainly occur in lymphocytes and not in oligodendrocytes in EAE [72]. The contribution of virus-induced oligodendrocyte apoptosis during the pathogenesis of TMEV-IDD is debated controversially, but generally regarded to be low for the TMEV BeAn-strain used in this study [27], [73], [74].

Conclusively, based on the provided results, MOG- and PLP-induced EAE as well as TMEV-IDD are suggested to mimic especially MS pattern II. In contrast, the TNFtg model displayed transcriptional changes anticipated to occur in MS pattern I.

The achievable level of novel information in a meta-analysis strongly depends on the availability and quality of raw data [10], [13], [75]. Therefore, the currently low number of publically available MS raw data sets despite multiple published microarray gene expression studies is a major handicap for the scientific community in this field of research. Cross-study GSEA outmatched the list comparisons and revealed interesting commonly affected pathways in MS, EAE, TMEV-IDD, and TNFtg including coagulation which represents a promising target for future studies. For unknown reasons we observed a lack of the anticipated transcriptional changes suggestive of oligodendrocyte dystrophy and apoptosis in TNFtg. Further microarray studies in toxin-induced animal models like cuprizone or ethidium bromide are needed to engage the hypothesis of multiple etiology and pathogenesis in MS on the transcriptional level.

## Supporting Information

Table S1 **List of 3788 genes with official Human Genome Organization (HUGO) gene symbols present on all five array platforms used in the multiple sclerosis (MS), experimental allergic encephalomyelitis (EAE), Theiler’s murine encephalomyelitis virus-induced demyelinating disease (TMEV-IDD) and transgenic tumor necrosis factor-overexpressing mouse (TNFtg) studies, which were included in the cross-study Gene Set Enrichment Analysis (GSEA).** Displayed are the log_2_-transformed and scaled expression values from all controls (n = 29) and all diseased individuals (n = 35). dpi = days post infection.
(XLSX)

Table S2 **List of differentially expressed genes in multiple sclerosis obtained by re-analysis of raw data from the study of Han et al. (2012).**
(XLSX)

Table S3 **List of differentially expressed genes in multiple sclerosis based on the studies of Lindberg et al. (2004) and Tajouri et al. (2003).**
(XLSX)

Table S4 **List of differentially expressed genes in myelin oligodendrocyte glycoprotein-induced experimental autoimmune encephalomyelitis obtained by re-analysis of the raw data from the study of Mueller et al. (2008).**
(XLSX)

Table S5 **List of differentially expressed genes in proteolipid protein-induced experimental autoimmune encephalomyelitis obtained by re-analysis of raw data from the study E-GEOD-44989.**
(XLSX)

Table S6 **List of differentially expressed genes in Theiler’s murine encephalomyelitis virus-induced demyelinating disease (TMEV-IDD) obtained by re-analysis of the raw data from the study of Ulrich et al. (2010).**
(XLSX)

Table S7 **List of differentially expressed genes in a transgenic tumor necrosis factor-overexpressing mice (TNFtg) obtained by re-analysis of the data from Tseveleki et al. (2010).**
(XLSX)

Table S8 **List of the intersections of differentially expressed genes in multiple sclerosis, experimental autoimmune encephalomyelitis, Theiler’s murine encephalomyelitis virus-induced demyelinating disease, and transgenic tumor necrosis factor-overexpressing mice displayed in** **Figure 2** **.** Red = positive fold change, Green = negative fold change, FC = fold change, q = false discovery rate.
(XLSX)

Table S9 **List of the genes comprised in the 21 commonly enriched gene sets in diseased versus control individuals of multiple sclerosis, experimental autoimmune encephalomyelitis, Theiler’s murine encephalomyelitis virus-induced demyelinating disease, and transgenic tumor necrosis factor-overexpressing mice revealed by Gene Set Enrichment Analysis (GSEA).** Red = positive fold change, Green = negative fold change, FC = fold change, q = false discovery rate.
(XLSX)

Table S10 **The gene signatures for the GO terms “T cell mediated immunity”, “immunoglobulin mediated immune response”, “positive regulation of apoptosis”, and “myelination” that are shown in** **Figure 4** **.** Displayed are the fold changes and q-values of these genes in experimental autoimmune encephalomyelitis, Theiler’s murine encephalomyelitis virus-induced demyelinating disease, and transgenic tumor necrosis factor-overexpressing mice, respectively. Red = positive fold change, green = negative fold change, FC = fold change, q = false discovery rate.
(XLSX)

Table S11 (DOC)

Checklist S1 **PRISMA Checklist.**
(DOCX)
